# Telemonitoring post-renal transplantation and role of advanced practice nurses: a single center experience

**DOI:** 10.3389/fneph.2026.1776371

**Published:** 2026-04-07

**Authors:** Delphine Bailly, Maher Abdessater, Ramy Touma Sawaya, Benoit Barrou, Sarah Drouin

**Affiliations:** 1 Department of Urology, Hopital Foch, Suresnes, France; 2 Division of Urology, Department of Surgery, American University of Beirut Medical Center, Beirut, Lebanon; 3 Academic Department of Urology of la Pitié-Salpêtrière Hospital, Assistance Publique-Hôpitaux de Paris, Faculté de Médecine Pierre et Marie Curie, University Paris VI, Hôpital Pitié, 47-83 bvd de l’Hôpital, Paris, France

**Keywords:** advanced practice nurse, healthcare outcomes, kidney transplantation, patient experience, telemonitoring

## Abstract

**Introduction:**

Telemonitoring has the potential to improve access to care and continuity of follow-up after kidney transplantation. Advanced practice nurses (APNs) play an increasingly important role in coordinating remote care pathways. This study evaluated patient experience with telemonitoring after renal transplantation, identified determinants of adherence, and clarified the role of APNs in this model.

**Methods:**

We conducted a single-center retrospective observational study including adult kidney transplant recipients enrolled in a telemonitoring program between April 2020 and April 2022. Patients were classified as active users (TOUCO), discontinued users (STOPCO), or never users (JAMCO). Satisfaction and experience were assessed through questionnaires. Platform activity and APN workload were analyzed using descriptive statistics.

**Results:**

Among 207 eligible patients, 110 responded to the survey (53%): 64 TOUCO (71%), 11 STOPCO (47%), and 35 JAMCO (37%). Active users reported high satisfaction with response time (89%), improved access to care (81%), and increased reassurance (75%). Ease of use (86%) and adequate information at enrollment were significantly associated with continued use. Major barriers included technical difficulties (≈80%) and loss of login credentials (>50%). During the study period, 5,214 platform events and more than 4,000 secure messages were recorded, reflecting sustained engagement. APNs required a mean workload of 3 hours per day to manage all active users on a daily basis.

**Conclusion:**

Telemonitoring after kidney transplantation is feasible and well accepted, improving perceived access to care and enhancing patient reassurance without measured clinical outcome differences. Adherence is driven primarily by organizational and technological factors rather than patient characteristics. APNs play a central role in ensuring continuity of care, triaging data, and maintaining patient engagement. Future studies should evaluate clinical outcomes and cost-effectiveness to support broader implementation.

## Introduction

Since 2012, the “Haute Autorité de Santé” (HAS), an independent public scientific body tasked with assessing drugs, medical devices, and procedures, as well as publishing guidelines and certifying healthcare organizations to improve care quality and equity (1), has considered kidney transplantation the treatment of choice for patients with end-stage renal disease (2). It improves the patient’s life expectancy and quality of life compared to dialysis, which on the contrary, impacts all areas of his daily life (3).

In response to the aging population, the increasing number of chronic pathologies, and the lack of specialists, the law for the modernization of the health system was published in 2016 (4, 5), and defined a new status of advanced practice nurse (APN). APNs specializing in nephrology, dialysis, and kidney transplantation, among other responsibilities, support doctors with the increasing number of kidney transplant patients. An APN can monitor stable patients and refer them to physicians when necessary (6, 7). This new reform intends to improve healthcare access and the quality of pathways, diversify the practice of paramedical professions, and progress to a high level of expertise (8, 9).

Telemedicine was defined by article L6316 of the public health code as a remote medical practice using information and telecommunication technologies (10). Its development and deployment have been among the government’s priority objectives as part of the national health strategy (11). One of the branches of telemedicine is telemonitoring, which consists of the remote medical interpretation of the data collected at the patient’s home to improve health (12, 13). For the HAS, telemonitoring allows regular follow-up and constitutes a significant path for enhancing the quality of care, increasing efficiency, and improving the quality of life by preventing complications. It is particularly suitable for chronic pathologies (14).

The “Expérimentations de Télémédecine pour l’Amélioration des Parcours en Santé” (ETAPES) program offered financial funding for the deployment of telemonitoring projects over a period of 4 years from 2018 to 2021. Renal failure was one of the five supported pathologies (15, 16). In France, telemedicine use took off in March 2020, with the onset of the COVID-19 pandemic. As the virus spread, solutions had to be found for precise, continuous monitoring of kidney transplant patients, even from long distances. Telemonitoring allowed this follow-up while avoiding direct patient contact and the high risk of contamination (17, 18).

The transplant center workflow is structured around a coordinated, multidisciplinary pathway that spans pre-transplant evaluation, surgical intervention, and long-term follow-up. Patients enter through the consultation unit, where resolute teams of nephrologists, urologists, anesthesiologists, and specialized nurses conduct comprehensive recipient and donor assessments to confirm eligibility. Approved candidates begin a planned hospitalization pathway, supported by inpatient nursing staff, transplant coordinators, psychologists, social workers, and allied health professionals who address medical, psychosocial, and logistical needs. The workflow integrates a hemodialysis unit staffed by nephrologists and critical care personnel for urgent and postoperative dialysis, as well as an intensive care unit led by anesthesiologist–intensivists and trained nurses to manage perioperative instability and transplant-related emergencies. Surgical procedures are performed in parallel operating rooms by specialized teams including experienced transplant surgeons, surgical residents, anesthesiologists, nurse anesthetists, scrub nurses, and operating room nurses. Across all units, the multidisciplinary transplant team, consisting of immunologists, rehabilitation specialists, dietitians, physiotherapists, and coordinators, ensures continuity of care, patient education, graft logistics, and long-term follow-up, supported by 24/7 laboratory and imaging services to deliver safe and efficient transplant care.

The APNs of our study play a significant role in coordinating care across the consultation, hospitalization, and hemodialysis units, allowing for continuous patient engagement and a comprehensive understanding of each patient’s clinical trajectory. They conduct pre- and post-transplant assessments, monitor clinical status, facilitate multidisciplinary communication, and ensure adherence to immunosuppressive regimens and follow-up protocols. Through patient education, early identification of complications, and coordination of transitions between care settings, APNs contribute to improved continuity of care, enhanced patient safety, and optimized transplant outcomes. APNs also autonomously managed protocol-driven tasks such as laboratory reminders, patient education, adherence counseling, and first-line triage of alerts. Medication adjustments within predefined ranges were proposed by APNs but required physician validation. Complex clinical decisions, graft dysfunction, or suspected rejection were escalated to transplant physicians. Their sustained presence throughout the transplant pathway provides unique insight into patients’ medical and psychosocial needs, positioning them as key contributors to individualized care and quality improvement initiatives.

Remote digital interventions in medicine have been shown to improve medication adherence, health status, and economic outcomes, reinforcing their value in long-term graft surveillance (19). When considering data on kidney failure and chronic kidney disease, telehealth interventions in kidney disease populations have demonstrated improvements in symptom burden, psychological well-being, and quality of life (20), and has shown clinical outcomes comparable to in-person care while reducing outpatient visits and healthcare costs (21). However, certain concerns have risen regarding telemedicine including limited internet access and difficulty mastering digital platforms, highlighting the importance of technical proficiency and infrastructure (22). For kidney transplants, a 2024 Randomized data analysis on dialysis and transplantation wards demonstrated that telemonitoring can safely reduce unplanned consultations by 26% and hospitalizations 32% while decreasing medical workload (22). When considering the effectiveness of APNs, many studies have shown that telehealth interventions led by APNs have demonstrated improvements in quality of life, self-management, and clinical indicators across chronic disease populations (23–25).

The objectives of this study were to assess patients’ experiences with telemonitoring, propose strategies to improve adherence, and clarify the role of the APN in the implementation of this novel counseling approach.

## Material and methods

### Study design and setting

This was a single-center, retrospective observational study conducted at the kidney transplant unit of our tertiary university hospital. The study aimed to assess patients’ experiences with telemonitoring after kidney transplantation, identify factors influencing adherence, and describe the role of APNs in this remote follow-up model. The study recruited patients who underwent kidney transplants between April 2020 and April 2022.

The study analyzed data collected during the predefined evaluation period; however, the telemonitoring application itself remains active and continues to be used in routine clinical practice at our institution.

### Participant training

At three months post-transplant, eligible patients were informed about the telemonitoring application by their nephrologist during routine follow-up visits. The eligible and accepting participants were then approached by the application representatives and technical support to explain the details of the application briefly, explain the risks and benefits of registering for the application, and offer an expedited overview on how to use the application. The participants were then invited, with their primary caregivers if needed, to an in-depth training session that was done once monthly. This training session was done by a specialized team consisting of technical support, APNs, and medical specialists that are familiar with the application, and involved hands-on use of the application on the participants’ preferred devices.

### Telemonitoring system

Telemonitoring was performed using the apTeleCare^®^ application (Tmm Software), which is accessible via computer, smartphone, or tablet. The application is approved by the French Ministry of Health, certified as a class I medical device, and complies with French health data hosting (HDS) regulations to ensure data security and patient confidentiality.

Through apTeleCare^®^, patients transmitted predefined clinical and biological data. Data transmission followed a predefined clinical schedule rather than patient-driven reporting. These include a twice monthly vital signs readings (heart rate, respiratory rate, blood pressure, weight/BMI, and oxygen saturation), regular blood tests including electrolytes, complete blood count, kidney function tests, and urinalysis if needed. The frequency of biological data collection depended on the time elapsed since transplantation and the patient’s clinical status. During the first post-transplant year, data were recorded monthly; thereafter, assessments occurred every 1–4 months according to patient stability, with more frequent evaluations for those with poorer clinical status. This structured schedule ensured standardized follow-up and limited variability in transmission frequency across adherent users.

A secure messaging system enabled asynchronous communication between patients and healthcare professionals for non-urgent issues, as fully explained during the initial consent process to register for the teleservice. Patients also had the option of submitting **Supplementary Material**, examinations and testing to their page for follow-up and monitoring.

Healthcare providers could review transmitted data, respond to messages, and send prescriptions when necessary. The healthcare team can also provide documents, satisfaction surveys, and research related material for patients to look into. All data exchanges were encrypted to maintain patient security and privacy from external parties.

APNs served as first-line reviewers of incoming data and alerts. They independently managed routine parameters within predefined protocols (e.g., medication titration, laboratory reminders) and escalated complex clinical decisions to transplant physicians. All prescriptions and major therapeutic adjustments required physician validation according to institutional policy. The APN would connect to the software daily in order to respond to inquiries, follow up on upcoming patient appointments, and gather the various new reports uploaded by the connected patients. The institutional target response time was within 24 hours on working days, except weekends and public holidays. Precise response-time metrics per message could not be retrospectively extracted from the platform; therefore, response time is reported as a policy target rather than a measured outcome. The APN also responded to alerts when transmitted information identified the possibility of a patient deteriorating or becoming unstable, such as with lab results, regular check-ins, or following certain examinations. This information was also shared with the referring and primary physicians in order to maintain strong and consistent medical care.

### Study population

All adult kidney transplant recipients enrolled in apTeleCare^®^ were eligible for inclusion. Inclusion criteria were age older than 18 years, kidney transplantation performed at least three months prior to enrollment, and agreement to register for the telemonitoring application.

Patients were excluded if they had documented cognitive impairment, were unable to communicate in French, lacked an email address, were registered for less than three months, or had a deactivated apTeleCare^®^ account at the time of the study (due to death, prolonged non-use, or technical issues).

Based on telemonitoring usage patterns, participants were classified into three predefined groups. The TOUCO group consisted of patients who used the application regularly and transmitted data according to medical recommendations. During the first post-transplant year, patients were expected to transmit clinical and biological data monthly; thereafter, transmission occurred every 1–4 months depending on clinical stability, with more frequent monitoring for unstable patients. This schedule corresponded to the standardized monitoring protocol described in the telemonitoring system section. This group received satisfaction surveys aimed at identifying the advantages of consistent telemonitoring. The STOPCO group included patients who initially used the application but discontinued data transmission after several months. This group received feedback questionnaires that aimed to identify the obstacles and hindrances to long-term use of the telemonitoring application. The JAMCO group consisted of patients who accepted and registered for telemonitoring follow-up but never logged into the application. This group received a separate questionnaire aimed at identifying the factors that initially attracted them to the application and the barriers that prevented them from registering. These categories were clinically relevant to distinguish sustained engagement, early discontinuation, and non-adoption of telemonitoring.

The TOUCO and STOPCO groups had an additional inclusion criterion to ensure proper group allocation: registration and use of the application for at least three months. The STOPCO and JAMCO groups received their medical information during follow-up visits only and filled the supplied questionnaires during the appointments or online when email reminders were dispersed. Because these groups were not highly connected, participants with uncompleted surveys were contacted via phone call three weeks after the reminder to request completion, if willing.

### Data collection and questionnaires

Data was collected using online questionnaires created with Microsoft Forms. Questionnaires included closed-ended questions and a limited number of open-ended questions to capture qualitative feedback. The questionnaires were not externally validated instruments, but rather locally developed for service evaluation. The questionnaires were tailored to each group to explore determinants of use, barriers to adherence, perceived benefits, and areas for improvement. The questionnaires were first distributed to all groups, and then the STOPCO and JAMCO groups received reminders 1 month after. Questionnaires were sent in early April 2022 and were closed in May 2022.

In addition, APNs involved in telemonitoring prospectively recorded the time spent managing the platform over seven consecutive days, including message responses, data review, and coordination of care.

### Variables

Collected variables included sociodemographic data (age, sex, educational level, professional status), transplant-related characteristics (number of transplantations, time since transplantation), and telemonitoring-related factors (quality of information at enrollment, ease of use, response time, perceived access to care, reassurance, autonomy, satisfaction, and self-reported technical proficiency). Technical proficiency was defined as the patient’s self-reported ability and confidence in using electronic devices and digital applications for health-related purposes and was indirectly assessed through questionnaire responses regarding technical difficulties, comfort with the platform, and need for assistance.

### Feasibility framework

This study evaluated feasibility across three predefined domains: (1) acceptability (patient satisfaction, continued platform engagement, and questionnaire responses), (2) implementation (onboarding procedures, nurse workflow integration, and alert management), and (3) practicality (data transmission frequency, nurse workload, and response time metrics). These domains were selected in line with standard feasibility evaluation frameworks for digital health interventions.

### Statistical analysis

Data analysis was performed using Plavue.io software. Descriptive statistics were used to summarize patient characteristics and questionnaire responses. Quantitative variables were expressed as means ± standard deviation or medians with interquartile ranges, as appropriate. Qualitative variables were expressed as frequencies and percentages.

Comparisons between groups were performed using the Wilcoxon test for quantitative variables and the chi-square test or Fisher’s exact test (when expected cell counts were <5) for qualitative variables. For some analyses, STOPCO and JAMCO participants were combined into a single “non-connected” (NOCO) group. A p-value <0.05 was considered statistically significant.

### Ethical considerations

The study was conducted in accordance with the principles of the Declaration of Helsinki. Participation was voluntary, and questionnaire responses were anonymous. All collected data were handled in compliance with French data protection regulations.

## Results

### Sociodemographic

A total of 207 questionnaires were distributed to all eligible patients registered on the application, 90 to TOUCO, 25 to STOPCO, and 95 to JAMCO, yielding 110 responses (53% overall). Responses were received from 64 (71%) TOUCO participants, 11 (47%) STOPCO participants, and 35 (37%) JAMCO participants. A substantial proportion of enrolled patients were part of the JAMCO group, reflecting early non-adoption rather than discontinuation after use. Sociodemographic characteristics are represented in **Table 1**. Response rates were higher among TOUCO than among STOPCO or JAMCO users. No significant differences were seen when comparing the sociodemographic variables between TOUCO and NOCO groups. The home-to-hospital distance didn’t affect the usage of the application, with 43.8% of the TOUCO group and 34.8% of the NOCO group living at least 30 km from the hospital (p=0.97).

**Table 1:** Sociodemographic characteristics of the three studied populations.

	n (%) or mean ± SD (Min-Max)				
Variable	All responders	TOUCO	STOPCO	JAMCO	NOCO
Total	110	64 (58.2)	11 (10)	35 (31.8)	46 (41.8)
Gender					
Male	70 (63.6)	37 (57.8)	10 (90.9)	23 (65.7)	33 (71.7)
Female	40 (36.4)	27 (42.2)	1 (9.1)	12 (34.3)	13 (28.3)
Age (years)	56 ± 12.7	57 ± 11.6	48 ± 10.5	56 ± 14.5	52 ± 14
Number of transplant					
1^st^	93 (84.5)	53 (82.8)	9 (81.8)	31 (88.6)	40 (87.0)
2^nd^	17 (15.5)	11 (17.2)	2 (18.2)	4 (11.4)	6 (13.0)
Graft term (years)	7 (6.4)	7 (10.9)	6 (54.5)	8 (22.9)	7 (15.2)
Professional status					
Retired	51 (46.4)	33 (51.6)	4 (36.4)	14 (40.0)	18 (39.1)
Student	3 (2.7)	0 (0.0)	1 (9.1)	2 (5.7)	3 (6.5)
Independent	11 (10.0)	9 (14.1)	1 (9.1)	1 (2.9)	2 (4.3)
Employee	45 (40.9)	22 (34.4)	5 (45.5)	18 (51.4)	23 (50.0)
Degree					
None	7 (6.4)	5 (7.8)	0 (0.0)	2 (5.7)	2 (4.3)
High school	15 (13.6)	7 (10.9)	2 (18.2)	6 (17.1)	8 (17.4)
CAP, BEP	27 (24.5)	14 (21.9)	4 (36.4)	9 (25.7)	13 (28.3)
University	61 (55.5)	38 (59.4)	5 (45.5)	18 (51.4)	23 (50.0)
Distance from hospital					
More than 30 km	44 (40.0)	28 (43.8)	4 (36.4)	12 (34.3)	16 (34.8)
Between 20 and 30 km	17 (15.5)	9 (14.1)	2 (18.2)	6 (17.1)	8 (17.4)
Between 10 and 20 km	20 (18.2)	11 (17.2)	3 (27.3)	6 (17.1)	9 (19.6)
Less than 10 km	29 (26.4)	16 (25.0)	2 (18.2)	11 (31.4)	13 (28.3)

TOUCO, always connected; STOPCO, stopped connection; NOCO, not connected; JAMCO, never connected; CAP BEP, professional diplomas.

**Figure 1:**
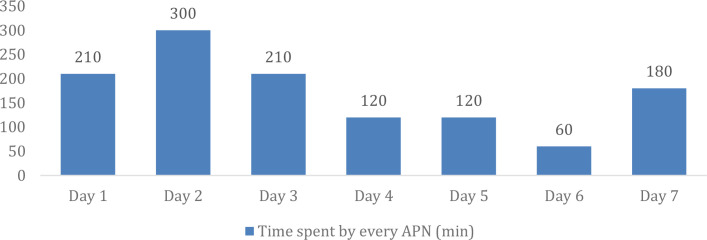
Average daily time spent by an APN managing the telemonitoring application for 90 patients, derived from pooled data across multiple APNs to reflect the typical daily workload.

The average time spent by APN on the application was 171.4 mins which is around 3 hours per day to manage 90 patients as seen in **Figure 1**. The average was taken for all the 90 TOUCO patients that were registered and fully connected as per recommended on the application.

### Events and communications

During the study period (April 2020–April 2022), a total of 5,214 application events were recorded, with peak activity in 2021 (2,987 events) compared with 1,475 in 2020 and 752 in early 2022. The majority of events consisted of blood and urine test submissions (52.6%), followed by medication orders (15.3%) and other events (12.0%). Additional functions included remote prescriptions (6.0%), tacrolimus level monitoring (3.8%), and pandemic-related activities such as COVID-19 serology (1.9%) and vaccination follow-up (1.4%). Communication through the platform remained substantial, with 2,301 messages sent by the medical team and 2,091 by patients, again peaking in 2021. Caregiver alerts were frequent, including 1,625 threshold alerts and 710 non-filling alerts, although non-filling alerts declined to zero by 2022. Overall, monthly and daily averages demonstrated sustained engagement, with the highest interaction rates observed in 2021 and stable utilization maintained into 2022. Given that TOUCO users adhered to scheduled transmissions, the high number of laboratory uploads and messages reflects protocol-driven monitoring rather than unscheduled patient-initiated contacts. Messaging activity remained substantial throughout the study period, reflecting continuous interaction between patients and the care team within the structured telemonitoring workflow. This information is summarized in **Table 2**.

**Table 2:** Application activity and communication metrics during the study period.

Outcomes	2020	2021	2022
	(April-December)		(January – April)
Total events (n=5214)			
Per year	1475	2987	752
Per month	160	248	150
Per day	13	12	7
Type of events for study duration n (%)			
Blood and urine tests	2740 (52.6)		
Medication orders	796 (15.3)		
Additional examination results	311(5.9)		
Tacrolimus level monitoring	200 (3.8)		
Remote prescriptions	313 (6.0)		
COVID-19 serology	101 (1.9)		
Vaccination follow up	75 (1.4)		
Letters from medical professionals	51 (1.0)		
Other events	627 (12.0)		
Messages sent by medical team (n=2084)			
Per year	754	1276	271
Per month	83	106	54
Per day	4	5	2.7
Messages sent by patients			
Per year	641	1179	271
Per month	71	98	54
Per day	3	5	2.7
Alerts RECEIVED BY caregivers			
BOUND Alert (threshold alert)			
Per year	633	834	158
Per month	70	70	31
Per day	3.5	3.5	1.5
Non-Filling Alert (data not filled in by the patient)			
Per year	414	296	0
Per month	46	24	0
Per day	2.3	1	0

Study period definition: 2020 data include April–December; 2022 data include January–April.

Event definition: Events refer to all patient-generated and system-recorded activities within the application, including laboratory uploads, prescriptions, alerts, and administrative documents.

Percentages: Percentages are calculated based on the total number of events recorded during the study period (N = 5,214).

Messages include secure in-application communications between patients and the medical team.

Bound alerts: triggered when submitted values exceed predefined clinical thresholds.

Non-filling alerts: generated when expected patient data were not submitted within the scheduled timeframe.

Rate calculations: Per-month and per-day values represent averages calculated over the active months within each reporting year.

### Satisfaction, training, and feedback

Information regarding the modality of telemonitoring was significantly associated with application use; 79% of the TOUCO group, compared with 67% of the NOCO group, reported being sufficiently informed about the telemonitoring application modalities (p = 0.012). Difficulty communicating with the healthcare team through messaging, sending lab results, or receiving prescriptions was also significantly associated with consistent application use; 38% of the STOPCO group versus 6% of the TOUCO group reported insufficient functionalities to facilitate communication (p = 0.012).

Response time influenced user satisfaction and, consequently, application use, with satisfaction rates of 89% in the TOUCO group and 63% in the STOPCO group (p < 0.03). Similar findings were observed regarding improved access to healthcare, with 81% of the TOUCO group reporting easier access through telemonitoring compared with 54% of the STOPCO group (p = 0.022). In addition, TOUCO participants were more likely to perceive the application as easy to use (86% vs. 64%, p = 0.003) and reported greater improvements in overall well-being related to disease management (61% vs. 18%, p = 0.019).

Application use enhanced patients’ sense of autonomy in managing their disease, with 69% in the TOUCO group versus 27% in the STOPCO group (p = 0.015), and increased reassurance, reported by 75% versus 27%, respectively (p = 0.015). Although trends favored TOUCO users for better understanding of pathology (50% vs. 18%), improved knowledge of treatment options (50% vs. 18%), and better recognition of worrisome signs and symptoms (52% vs. 18%), these differences did not reach statistical significance.

Overall, users in the TOUCO group rated telemonitoring more favorably than those in the STOPCO group (mean scores 7.36 vs. 4.55 out of 10), and a substantially higher proportion recommended its use to others (94% vs. 55%). This information is summarized in **Table 3**.

**Table 3:** Self-reported perceptions and satisfaction by group.

Outcome	TOUCO (n = 64)	STOPCO (n = 11)	p-value
Believe the application is easy to use	55 (85.9)	7 (63.6)	**0.003**
Insufficient communication functionalities	4 (6.3)	4 (36.4)	**0.012**
Satisfied with response time	57 (89.1)	7 (63.6)	**<0.03**
Improved access to healthcare	52 (81.3)	6 (54.5)	**0.022**
Improved sense of well-being in management	39 (60.9)	2 (18.2)	**0.019**
Felt more autonomous in disease management	44 (68.7)	3 (27.3)	**0.015**
Allowed better understanding of pathology	32 (50)	2 (18.2)	0.056
Improved knowledge on treatment and options	32 (50)	2 (18.2)	0.14
Better recognition of worrisome signs and symptoms	33 (52)	2 (18.2)	0.13
Felt more reassured	48 (75)	3 (27.3)	**0.015**
Would recommend telemonitoring	60 (93.7)	6 (54.5)	—

Data are presented as number (percentage) of patients within each group.

Bolded p-values <0.05 are considered statistically significant.

JAMCO group users reported different reasons for not using the application, varying from the lack of connection between hospitals and the fear of privacy breaching. Some users also noted that the application was not downloadable. TOUCO group users highlighted the advantages of using the application being a reactive time-saving communication method, allowing continuous follow-up. They also had several suggestions for improvement on the technical, messaging, and data collection levels. Patients in the STOPCO group stated that they stopped using the application due to the lack of ergonomics and the absence or delay in response time. Additionally, they proposed synchronizing it with the Shared Medical Record, also known as “Dossier Médical Partagé” (DMP). The DMP is a shared, nationally protected, medical recording system that is accessible by all patient-involved healthcare professionals in medical centers, whether academic or not, in France. Open-ended questions and answers are presented in **Table 4**.

**Table 4:** Answers to open-ended questions grouped by subgroups.

TOUCO	
Suggestions for improvement	
Technical	· Adjust smartphone and tablet access · Adjust access to prescriptions and documents
Data collection	· Improve data upload · Improve results transmission · Increase the ability to transfer results
Messaging	· Feedback for messages delivery · Notifications system · Improve the response delay · Hotline for emergency and fast responses · Results analysis by a doctor
Benefits of connection	
Communication	· Documents exchange · Reactivity · Fast response
Follow up	· Continuous lab results transmission · Permanent follow up · Faster communication
Timesaving	· Feeling more monitored · Feeling more secure by always having the possibility of reaching someone · Feeling close to the department
STOPCO	
What was missing	· Ergonomics · Response to asked questions
Suggestions for improvement	· Synchronization with the shared medical record
JAMCO	
Privacy issues	· Loss of clinical control and direct clinical contact
What was missing	· The application was not downloadable · Interoperability between hospitals

Among respondents contacted after questionnaire resending, internet or technical difficulties were reported by 81.8% of STOPCO patients and 80.0% of JAMCO patients. Loss of login credentials affected 54.5% of STOPCO and 51.4% of JAMCO respondents. Requests for activation code re-sending at the time of survey contact were noted in 27.3% of STOPCO patients and 45.5% of JAMCO patients. Concerns regarding data protection were reported by 27.3% and 31.4% of STOPCO and JAMCO respondents, respectively. Additionally, 27.3% of patients in both groups indicated they were no longer interested and requested deactivation. Fear of reduced physician contact was reported by 36.4% of STOPCO and 34.3% of JAMCO patients, while 36.4% and 37.1%, respectively, considered the application to be very time-consuming. Finally, 63.6% of STOPCO and 62.9% of JAMCO respondents stated that they had not accepted registration merely to satisfy their physician. This information has been summarized in **Table 5**.

**Table 5:** Collected data from STOPCO and JAMCO users after questionnaire reminders.

Outcomes	STOPCO n (%)	JAMCO n (%)	
Total	11	33	
Responses			
Lost login credential	6 (54.5)	18 (51.4)	
Requested re-activation code at survey contact	3 (27.3)	15 (45.5)	
We’re not interested and asked for deactivation	3 (27.3)	9 (27.3)	
Concerned about data protection	3 (27.3)	11 (31.4)	
Accepted registration just to satisfy physician	7 (63.6)	22 (62.9)	
Faced internet and technical Issues	9 (81.8)	28 (80)	
Feared reduction of physician contact	4 (36.4)	12 (34.3)	
Considered the application very time-consuming	4 (36.4)	13 (37.1)	

Values are presented as n (%) within each group.

Percentages are calculated using the total number of respondents in each group as the denominator.

Internet and technical issues include connectivity problems, device difficulties, or challenges using the application interface.

## Discussion

This single-center study identified key determinants of patient adherence to telemonitoring after kidney transplantation and highlighted the pivotal role of APNs in sustaining effective remote follow-up. Our findings indicate that adherence is primarily influenced by organizational and technological factors, such as quality of information, usability of the platform, and responsiveness of healthcare professionals, rather than by patient sociodemographic characteristics. These results are consistent with evidence showing that clarity of purpose, perceived usefulness, and system performance are critical drivers of engagement in telehealth interventions for chronic diseases (26). From a feasibility perspective, our findings support the acceptability, implementation, and practicality of telemonitoring in post-transplant care. Acceptability was demonstrated by high satisfaction, reassurance, and recommendation rates among active users. Implementation feasibility was reflected in the successful integration of telemonitoring into routine workflows, with APNs effectively triaging alerts and maintaining timely communication. Practicality was supported by sustained data transmission, manageable APN workload, and response times compatible with routine clinical practice. Together, these findings suggest that telemonitoring can be realistically integrated into transplant follow-up while maintaining care quality and patient engagement.

Our findings are consistent with transplant-specific telemonitoring studies, which report high patient satisfaction and improved perceived access to care without clear evidence of improved graft outcomes. Previous programs have demonstrated reductions in unplanned visits and enhanced patient reassurance, while emphasizing that organizational factors and professional responsiveness are primary determinants of sustained engagement rather than patient demographics (22–25).

Adequate patient information emerged as a major facilitator of telemonitoring use. Patients who clearly understood the objectives and modalities of telemonitoring were more likely to remain active users. Misunderstandings between telemonitoring and teleconsultation were frequently reported among non-users, emphasizing the need for structured educational materials. Prior studies have demonstrated that insufficient explanation of digital health tools reduces adoption and long-term engagement, regardless of patients’ age or education level (27). These findings support the implementation of standardized information sheets and inclusion protocols at the time of enrollment. Ease of use and short response times were also strongly associated with patient satisfaction and continued utilization. Timely responses to patient messages improved perceived access to care and reinforced feelings of reassurance and safety. Continuous interaction with healthcare professionals has been shown to enhance patient confidence and trust in remote care models, particularly in chronic disease settings requiring frequent biological monitoring (27). In our cohort, the ability to transmit laboratory results was the most valued feature, underscoring the central role of biological surveillance in post-transplant care.

Despite these benefits, telemonitoring did not significantly improve patients’ knowledge of their disease, treatments, or warning signs. This finding is consistent with previous literature demonstrating that remote monitoring alone does not replace structured therapeutic education (28). The absence of embedded educational resources within the application likely contributed to this result. Integrating targeted educational tools, such as guidance on immunosuppressive therapy, infection prevention, vaccinations, lifestyle recommendations, and travel precautions, may enhance patient empowerment and self-management.

Two major barriers to telemonitoring adoption were identified: loss of login credentials and limited technical proficiency. Technical proficiency refers to the ability to effectively use, understand, and apply technology, tools, or specialized systems to perform tasks accurately and efficiently, and was self-reported in this study as the ability and confidence in using technology and electronic devices on a regular basis. These obstacles are widely reported in telehealth research and contribute significantly to non-use or discontinuation of digital health tools (29). Even among patients who received initial training, discomfort with information technology remained a limiting factor. The establishment of a dedicated hotline for patients’ nonmedical concerns and technical issues, and personalized training sessions on application use and maintenance, potentially involving IT experts, caregivers, and other more experienced patients, could help overcome these barriers and promote inclusivity.

The relatively high proportion of JAMCO patients, representing early non-adoption of the platform, highlights a well-recognized challenge in digital health implementation. Non-adoption may reflect a combination of factors, including limited technical proficiency, lack of perceived need among clinically stable patients, preference for traditional follow-up, and logistical barriers such as password loss or connectivity issues. In our cohort, the telemonitoring program was offered during routine care rather than targeted to high-risk or highly motivated patients, which may have contributed to lower initial uptake. Similar patterns of early non-engagement have been reported in telehealth programs, underscoring that adoption is influenced more by perceived usefulness and usability than by demographic or clinical characteristics.

No specific patient profile predictive of telemonitoring adherence was identified. Age, gender, educational level, professional status, and graft characteristics were not associated with sustained use. This suggests that telemonitoring can be broadly offered to kidney transplant recipients without restrictive selection criteria, provided adequate technical and organizational support is ensured. Similar conclusions have been reported in studies emphasizing that patient experience and system design are more influential than demographic characteristics alone (30).

The role of APNs was central to the success of the telemonitoring program. APNs ensured continuity of care, triaged incoming data, and responded to patient messages in a timely manner while managing a substantial virtual patient load. Evidence from systematic reviews supports the safety, efficiency, and quality of APN-led care in chronic disease management, including coordination, patient satisfaction, and optimization of healthcare resources. We propose a three-level alert system as a conceptual framework to optimize telemonitoring workflows. The proposed three-level alert system may further enhance efficiency by aligning clinical complexity with the appropriate level of professional expertise (31). At the first level, unspecialized nurses manage the first response to messaging that does not require essential decision-making or prescriptions. The second level is managed by the highly trained APNs who can give advice and medical knowledge on the basis of their competency framework. Finally, the third level consists of physicians who respond to highly specialized medical questions, especially those that revolve around continuation of treatment and conduction of diagnostic tests that, with the advice of the APNs, may be deemed necessary or urgent. Furthermore, the third level may be deemed necessary when patients no longer fit the stability criteria of the establishment, prompting essential healthcare from multidisciplinary teams of physicians and nurses. This three-level alert system can be used efficiently with telemonitoring applications such as the one proposed as it can allow for speedy responses by specialized personnel and assist in prioritizing patient care in emergency situations even if not present at the medical center or emergency department.

This study has several strengths. First, it provides a comprehensive assessment of telemonitoring after kidney transplantation by including not only active users but also patients who discontinued or never connected to the platform. This three-group approach allowed the identification of both facilitators and barriers to telemonitoring adoption, offering a more nuanced understanding of patient experience than studies focusing exclusively on adherent users. Second, the mixed-methods design, combining quantitative analysis with open-ended questions, enriched the interpretation of results by capturing patients’ perceptions, expectations, and practical difficulties that are not always detectable through structured questionnaires alone. Third, the study highlights the operational role of APNs in real-life telemonitoring, including workload estimation and care coordination, providing pragmatic data that are directly applicable to clinical organization and health system planning. Finally, the relatively high overall response rate supports the relevance of the findings and reflects patient engagement with the topic.

Importantly, the telemonitoring program was not discontinued at the end of the study period and remains operational in routine clinical practice. The predefined study timeframe reflects the institutional evaluation protocol rather than termination of the intervention. Lessons learned from this implementation include the importance of structured onboarding, technical support, and clear communication of the platform’s benefits to patients. Sustainable telemonitoring programs require integration into existing workflows, dedicated nursing coordination, and institutional commitment to digital infrastructure. Demonstrating the feasibility and patient acceptance of telemonitoring is a critical step toward informing health policy and resource allocation. Investment in telemonitoring may be justified by its potential to improve access to care, optimize professional workload, and enhance patient reassurance, particularly for populations requiring long-term follow-up. Future cost-effectiveness analyses and multicenter studies are needed to support broader reimbursement and national implementation strategies.

Several limitations should be acknowledged. The single-center design may limit the generalizability of the results to other transplant centers with different organizational structures, patient populations, or telemonitoring tools. The retrospective nature of the study and reliance on self-reported, non-validated questionnaires expose the findings to recall and response biases, particularly among patients who discontinued or never used the application. In addition, the small size of the STOPCO subgroup limited the statistical power of certain comparisons and prevented robust multivariable analyses. However, this limitation is common in feasibility and implementation studies of digital health interventions and should be considered when interpreting the results (32). Some questionnaire items, particularly those assessing digital accessibility and technical difficulties, could have been more detailed and may warrant refinement in future studies. Also, the statistical analysis was primarily descriptive, consistent with feasibility study objectives. No multivariable modeling or effect size estimation was performed. Therefore, associations between patient characteristics and platform engagement should be interpreted cautiously and cannot imply causality. Importantly, this study did not evaluate clinical outcomes such as graft function, rejection episodes, or hospitalization rates. Conclusions are therefore limited to feasibility, workflow integration, and patient-reported experience. As some information, such as the number of messages relayed and the types of events occurring, was extracted from the application itself, a major limitation was the inability to divide the information into the study’s different population groups (TOUCO, STOPCO, and JAMCO). Furthermore, considering the large number of messages and the lack of division, data on response time and drop-out time was not adequately collected and analyzed. Detailed attrition timelines and Kaplan–Meier analysis were not feasible due to unavailable time-stamped usage data. Technical proficiency was self-reported and not assessed using a validated instrument, which may limit measurement precision. The platform provided aggregate activity metrics but did not allow extraction of per-patient transmission frequency or weekly averages, limiting detailed analysis of individual engagement patterns. Detailed response-time metrics could not be extracted retrospectively from the telemonitoring platform. Although institutional policy targeted responses within 24 hours, the absence of precise measurements prevented analysis of response time as a determinant of adherence. The platform provided aggregate activity metrics but did not allow extraction of per-patient transmission frequency or weekly averages, limiting detailed analysis of individual engagement patterns. Response bias may have occurred, as active users were more likely to complete questionnaires, potentially overestimating satisfaction and perceived benefits.

Despite these limitations, the study provides valuable insights into the determinants of telemonitoring adherence after kidney transplantation and underscores the central role of advanced practice nurses in ensuring effective, patient-centered remote follow-up.

## Conclusion

Telemonitoring represents a valuable adjunct to conventional follow-up after kidney transplantation, improving access to care and enhancing patients’ sense of reassurance and well-being. This study shows that adherence to telemonitoring is primarily driven by organizational and technological factors rather than patient sociodemographic characteristics. Clear information at enrollment, ease of use of the digital platform, and rapid responses from healthcare professionals are key determinants of sustained patient engagement.

Despite high satisfaction among active users, telemonitoring alone did not significantly improve patients’ knowledge of their disease or treatments, highlighting the need to integrate structured educational resources within telemonitoring tools. Barriers such as limited digital proficiency and technical difficulties remain important challenges and should be addressed through tailored training, technical support, and simplified access procedures.

Advanced practice nurses play a central role in the success of telemonitoring programs by ensuring continuity of care, triaging patient data, and responding efficiently to patient needs. The implementation of a structured, tiered organizational model involving nurses, APNs, and physicians may further optimize workflow, safety, and scalability. Ongoing use of this telemonitoring platform in routine practice provides an opportunity to evaluate long-term outcomes and may inform the expansion of telehealth initiatives at national and international levels.

Overall, telemonitoring can be broadly offered to kidney transplant recipients without restrictive selection criteria, provided that adequate technical support and professional responsiveness are ensured. Future multicenter studies incorporating clinical outcomes and cost-effectiveness analyses are needed to better define the long-term impact of telemonitoring and to support its sustainable integration into post-transplant care pathways.

## Data Availability

The original contributions presented in the study are included in the article/**Supplementary Material**. Further inquiries can be directed to the corresponding author.
