# Supplementary material for: Telemonitoring post-renal transplantation and role of advanced practice nurses: a single center experience
Source: Front Nephrol. 2026 Apr 7;6:1776371. doi: 10.3389/fneph.2026.1776371 (PMC13104594; doi:10.3389/fneph.2026.1776371)
Supplement: Supplementary file 2 [file Table2.docx]

**Supplementary Material 2: Feedback questionnaire for STOPCO patients**

"*Dear Madam, Sir,*

*In collaboration with Prof. Barrou, I am carrying out a research work on monitoring by remote monitoring.*

*You use the apTeleCare app little or have stopped using it. We would like to hear your feedback on the difficulties you may have encountered.*

*Below is a link to complete a satisfaction survey, it will take less than 10 minutes. Responses will be anonymous.*

*Your participation is very important! It will allow us to develop this new way of monitoring.
Please return it to us before May 22, 2022.*

*Sincerely, Delphine Bailly.* »

- 1- I am:
- Male
- Female
- 2- My age is: .... (years)
- 3- This is my 1st transplant:
- Yes
- No
- 4- I have been transplanted for (in years if more than a year, in months if less than a year):...... (months/years)
- 5- My professional situation is:
- Employed
- Self-employed
- Student, in training, intern
- Retired or unemployed
- 6- The highest degree I have obtained:
- No diploma
- CAP, BEP, or equivalent diploma
- LAC
- Higher level diploma
- 7- How far do you live from the Pitié Salpêtrière hospital?
- Less than 10 kilometers
- Between 10 and 20 kilometers
- Between 20 and 30 kilometers
- More than 30 kilometers
- 8- I use the application on:
- A smartphone
- A computer
- A tablet
- I do not have computer equipment
- 9- I have difficulties connecting to the internet:
- Yes
- No
- 10- I have lost my access codes:
- Yes
- No
- 11- I have been sufficiently informed about the modalities of monitoring by remote monitoring:
- Totally agree
- Okay
- Disagree
- Strongly disagree
- 12- I have been trained in the use of the application:
- Yes
- No
- 13- A friend or family member helps me to use the application:
- Yes
- No
- 14- I think the app is difficult to use:
- Totally agree
- Okay
- Disagree
- Strongly disagree
- 15- I initially used the app to please my doctor:
- Totally agree
- Okay
- Disagree
- Strongly disagree
- 16- I am afraid that my medical data is not protected:
- Totally agree
- Okay
- Disagree
- Strongly disagree
- 17- I don't trust the application:
- Totally agree
- Okay
- Disagree
- Strongly disagree
- 18- I think that using the application is taking me too long:
- Totally agree
- Okay
- Disagree
- Strongly disagree
- 19- My medical data is easily accessible:
- Totally agree
- Okay
- Disagree
- Strongly disagree
- 20- I can easily enter my following medical data:
- My weight
- My blood pressure
- My laboratory assessment results
- None of the above
- 21- I find that communication with my healthcare team is facilitated by:
- The transmission of my biological results
- The chat system
- Sending prescriptions
- None of the above
- 22- I find the response time satisfactory when I contact my healthcare team via the application:
- Totally agree
- Okay
- Disagree
- Strongly disagree
- 23- Monitoring by remote monitoring improves my access to care:
- Totally agree
- Okay
- Disagree
- Strongly disagree
- 24- Thanks to the use of the application, I know my pathology better:
- Totally agree
- Okay
- Disagree
- Strongly disagree
- 25- Thanks to the use of the application, I know my treatments better:
- Totally agree
- Okay
- Disagree
- Strongly disagree
- 26- Thanks to the use of the application, I know better the signs that should alert me:
- Totally agree
- Okay
- Disagree
- Strongly disagree
- 27- Thanks to the application, I feel more autonomous in the management of the disease:
- Totally agree
- Okay
- Disagree
- Strongly disagree
- 28- Using the app has improved my sense of well-being:
- Totally agree
- Okay
- Disagree
- Strongly disagree
- 29- The use of the application reassures me:
- Totally agree
- Okay
- Disagree
- Strongly disagree
- 30- I recommend follow-up by remote monitoring to kidney transplant patients:
- Totally agree
- Okay
- Disagree
- Strongly disagree
- 31- What overall rating would you give to your remote monitoring follow-up:

| 0 | 1 | 2 | 3 | 4 | 5 | 6 | 7 | 8 | 9 | 10 |
| --- | --- | --- | --- | --- | --- | --- | --- | --- | --- | --- |

Not at all satisfied Extremely satisfied

- 32- What did you miss to continue your monitoring by remote monitoring?

…………………………………………………………………………………………………………………………

- 33- What are your suggestions for improvement?

………………………………………………………………………………………………………………………….
