# Supplementary material for: Telemonitoring post-renal transplantation and role of advanced practice nurses: a single center experience
Source: Front Nephrol. 2026 Apr 7;6:1776371. doi: 10.3389/fneph.2026.1776371 (PMC13104594; doi:10.3389/fneph.2026.1776371)
Supplement: Supplementary file 3 [file Table3.docx]

**Supplementary Material 3: Questionnaire for JAMCO patients who agreed to follow-up but never logged in**

*"Dear Madam, Sir,*

*In collaboration with Prof. Barrou, I am carrying out a research work on monitoring by remote monitoring.*

*You agreed to use the apTeleCare app but never logged in. We would like to hear your feedback on the difficulties you may have encountered.*

*Below is a link to complete a questionnaire, it will take you less than 10 minutes. Responses will be anonymous.*

*Your participation is very important! It will allow us to improve your care.
Please return it to us before May 22, 2022.*

*Sincerely, Delphine Bailly. »*

- 1- I am:
- Male
- Female
- 2- My age is: .... (years)
- 3- This is my 1st transplant:
- Yes
- No
- 4- I have been transplanted for (in years if more than a year, in months if less than a year):...... (months/years)
- 5- My professional situation is:
- Employed
- Self-employed
- Student, in training, intern
- Retired or unemployed
- 6- The highest degree I have obtained:
- No diploma
- CAP, BEP, or equivalent diploma
- LAC
- Higher level diploma
- 7- How far do you live from the Pitié Salpêtrière hospital?
- Less than 10 kilometers
- Between 10 and 20 kilometers
- Between 20 and 30 kilometers
- More than 30 kilometers
- 8- I use the application on:
- A smartphone
- A computer
- A tablet
- I do not have computer equipment
- 9- I have difficulties connecting to the internet:
- Yes
- No
- 10- I have been sufficiently informed about the procedures for monitoring by remote monitoring:
- Totally agree
- Okay
- Disagree
- Strongly disagree
- 11- I have been trained in the use of the application:
- Yes
- No
- 12- I think the app is difficult to use:
- Totally agree
- Okay
- Disagree
- Strongly disagree
- 13- I gave my agreement to please my doctor:
- Totally agree
- Okay
- Disagree
- Strongly disagree
- 14- I am afraid that my medical data is not protected:
- Totally agree
- Okay
- Disagree
- Strongly disagree
- 15- I don't trust the application:
- Totally agree
- Okay
- Disagree
- Strongly disagree
- 16- I think that using the application is taking me too long:
- Totally agree
- Okay
- Disagree
- Strongly disagree
- 17- I have lost my access codes:
- Yes
- No
- 18- I'm afraid I won't see my doctor and my healthcare team anymore:
- Totally agree
- Okay
- Disagree
- Strongly disagree
- 19- I don't have help from a family member to help me use the app
- Totally agree
- Okay
- Disagree
- Strongly disagree
- 20 – What attracted you initially to make you want to use remote monitoring?

…………………………………………………………………………………………………………………
